# Investigation Around Cases of Crimean-Congo Hemorrhagic Fever—Mauritania, 2022

**DOI:** 10.1093/ofid/ofac534

**Published:** 2022-10-12

**Authors:** Boushab Mohamed Boushab, Pauline K Yanogo, Djibril Barry, Hacen Ahmed Benane, Ahmed El Bara, Moussa Abdellah, Leonardo K Basco, Nicolas Meda

**Affiliations:** Department of Internal Medicine and Infectious Diseases, Kiffa Hospital Center, Kiffa, Assaba, Mauritania; Burkina Field Epidemiology and Laboratory Training Program, Université Joseph Ki-Zerbo, Ouagadougou, Burkina Faso; Burkina Field Epidemiology and Laboratory Training Program, Université Joseph Ki-Zerbo, Ouagadougou, Burkina Faso; Burkina Field Epidemiology and Laboratory Training Program, Université Joseph Ki-Zerbo, Ouagadougou, Burkina Faso; Burkina Field Epidemiology and Laboratory Training Program, Université Joseph Ki-Zerbo, Ouagadougou, Burkina Faso; Direction des Services Vétérinaires, Ministère de l'Elevage, Nouakchott, Mauritania; Laboratory of Virology, Institut National de Recherche en Santé Publique, Nouakchott, Mauritania; Direction de l’information Strategique et de la Surveillance Epidémiologique, Ministère de la Santé, Nouakchott, Mauritania; Aix-Marseille Université, IRD, AP-HM, SSA, VITROME, Marseille, France; IHU-Méditerranée Infection, Marseille, France; Burkina Field Epidemiology and Laboratory Training Program, Université Joseph Ki-Zerbo, Ouagadougou, Burkina Faso

**Keywords:** arbovirus, Crimean-Congo hemorrhagic fever, Mauritania

## Abstract

**Background:**

Crimean-Congo hemorrhagic fever (CCHF) is a zoonotic arbovirosis. Humans are infected by tick bites or contact with blood of infected animals. CCHF can be responsible for severe outbreaks due to human-to-human transmission. Our aims were to increase awareness and promote the search for risk factors and disease monitoring to prevent CCHF epidemic, capacity building, appropriate measures to treat patients, and information for the local population.

**Methods:**

During the outbreak of hemorrhagic fever from February to May 2022, blood samples were collected from 88 patients suspected to be infected with the virus. Diagnosis was established by reverse-transcription polymerase chain reaction (RT-PCR) and/or enzyme-linked immunosorbent assay.

**Results:**

CCHF was confirmed by RT-PCR in 7 of 88 (8%) patients. Ticks were found in cattle, sheep, or goats in the areas where the subjects resided, with the exception of 1 CCHF-positive patient in close contact with fresh animal meat. Exposure to potential risk factors was found in all patients. The interval between the onset of symptoms and hospital admission was 2–3 days. All 7 patients were admitted to our hospital and treated promptly by blood transfusion. Two patients died.

**Conclusions:**

Mortality is high in patients with the hemorrhagic form of CCHF. Disease prevention is necessary by strengthening vector control, avoiding contact and consumption of organic products from diseased animals, and vaccinating animals in areas where the disease is endemic. Furthermore, it is essential to establish management procedures for patients infected with CCHF virus.

Crimean-Congo hemorrhagic fever (CCHF) is a severe tick-borne disease, endemic in many countries in Africa, the Middle East, Eastern Europe, and Asia [1]. The etiological agent, CCHF virus (CCHFV), can be transmitted by argasid and ixodid ticks, but arachnids of the genus *Hyalomma*, followed by *Rhipicephalus* and *Dermacentor*, serve as the major vectors of this virus [2]. The virus is transmitted to humans through tick bites or direct contact with blood, secretions, or infected tissue of a viremic animal or person. The incubation period in humans is usually 5–6 days, and hemorrhage often occurs on the fourth or fifth day after the onset of illness. About 30% of patients die [3, 4].

This arboviral disease was seen for the first time in 1944 [4] in the south of present-day Ukraine and thus named Crimean fever. In 1956, the virus was isolated from a patient residing in Congo, with similar symptoms as those seen in Crimea, and the virus was named Congo virus [5]. CCHFV is highly divergent and presents the widest geographic range among all the medically important tick-borne viruses. Cases have been described in several areas of Africa, Asia, and Europe [6, 7]. In Mauritania, CCHF was first documented in 1983 [8]. Since that date, serological studies on human and animal cases have indicated a continued low-level circulation of CCHFV and transmission to humans from the animal reservoir (cattle, goats, sheep, and camels) in this region, particularly in southern and southeastern Mauritania [6, 8–12]. Considering the zoonotic nature, importance for public health, and the increasing spread of the virus in the country, the seroprevalence of CCHFV was assessed in 2015 in southeastern Mauritania where CCHF has been reported to be endemic [12]. Since the seroepidemiological survey in the country has been launched, the first case was confirmed on 4 February 2022, at the Kiffa Hospital Center in Kiffa, southern Mauritania, followed by other cases in Nouakchott, the capital of the country (Centre Hospitalier National de Nouakchott, Centre Hospitalier de l’Amitié, Centre Hospitalier Cheickh Zayed), and in Boutilimit (Hôpital Hamed de Boutilimit).

The present investigation was performed to monitor and confirm the resurgence of CCHF cases in 3 regions (locally called “wilaya”): Hodh El Gharbi (Tintane and Koubeni departments [called “moughtaa” locally]), Trarza (Ouad Naga, R’Kiz, and Boutilmit departments), and Nouakchott (Dar naim district). The purpose of this study is to increase awareness and promote (1) determination of risk factors in order to propose preventive measures by establishment of an active surveillance system; (2) capacity building of laboratory diagnosis; (3) implementation of procedures for the management of patients infected with CCHFV with the designation of a referral hospital; and (4) information for the local population.

## METHODS

### Study Site

The Islamic Republic of Mauritania is a country in West Africa, opening to the west on the Atlantic Ocean, to the northwest by Western Sahara, to the north by Algeria, to the east and southeast by Mali, and to the southwest by Senegal. The country includes a territory of >1 million km^2^ with a very low population density (3.87 inhabitants/km^2^). The Human Development Index in 2019 was 0.546, representing a global ranking of 157 [13]. Its position between the 15th and 27th degrees north latitude makes it a country of contact and transition between the Saharan desert (70% of the territory), to the north, and the Sahelian steppes, to the south.

### Subjects and Methods

Between February and May 2022, 8 patients were admitted to Centre Hospitalier de Kiffa (Kiffa department/Assaba region), Hôpital Hamed (Boutilimit department/Trarza region), Centre Hospitalier Cheikh Zeid, Centre Hospitalier National de Nouakchott, or Hôpital de l’Amitié (Nouakchott) with an initial clinical diagnosis of CCHF. This period of the year is characterized by a hot, dry season and transhumance of livestock, which favors the spread of ticks [11, 14]. The study population of the present study was individuals who were potentially in contact with these 8 patients suspected to be infected with CCHFV. The subjects were composed of farmers, breeders, butchers, family members, or close relatives in contact with the patients, and hospitalized patients. The search for other suspected cases was carried out in the health structures having received the initial 8 suspected cases.

This research included a review of medical records between February and May 2022 and an interview with health staff. For a suspected case who was identified and whose home could be found, an active search for the case was carried out in their place of residence. If the suspected case was present at the time of the investigation, inclusion in the investigation was proposed to him or her and to the members of the entourage. If the suspected case was absent or deceased, inclusion was proposed to members of his or her entourage. In the localities visited (ie, villages of the cases), a search for suspected or probable cases in the community was systematically carried out by questioning the people present.

A “suspected case” was defined as anyone presenting with a sudden onset of high fever and flu-like syndrome, unresponsiveness to appropriate antimalarial drugs, and presence (or absence) of signs of bleeding, such as gingival hemorrhage, injected conjunctivae, epistaxis, petechiae, bloody diarrhea or melena, and hematemesis. A “confirmed case” was defined as any suspected case, deceased or not, for whom the blood sample was confirmed CCHFV-positive by the reference laboratory (presence of immunoglobulin M [IgM] or the CCHF viral genome). A “probable case” was defined as any suspected case who died with hemorrhage before biological confirmation was carried out. A “contact” was defined as family members and neighbors living in close contact with a confirmed, suspected, or probable case with risk of exposure to CCHFV. These case definitions were based on the World Health Organization Ebola case definitions [15, 16].

Clinically, CCHF poses a problem of differential diagnosis with other arboviruses. Biological confirmation of arboviruses was carried out by the laboratory of the Institut National de Recherche en Santé Publique. The diagnosis of CCHF infection was confirmed by either the detection of the viral genome by reverse-transcription polymerase chain reaction (RT-PCR) or by the demonstration of specific IgM antibodies by enzyme-linked immunosorbent assay (ELISA). For each patient, IgM antibodies against the following viruses were also tested in addition to CCHFV: yellow fever, West Nile, dengue fever, and chikungunya.

## RESULTS

**Figure 1.**
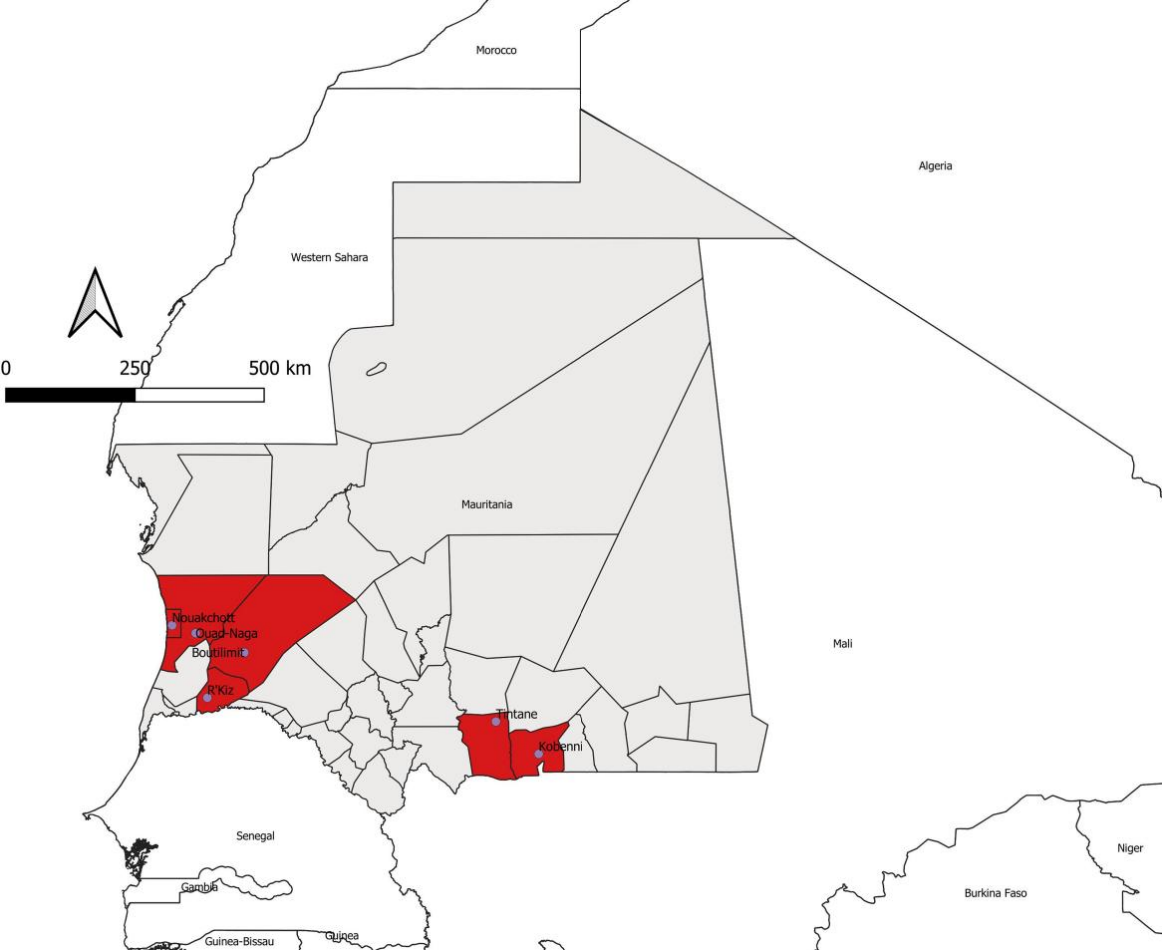
Geographic distribution of confirmed cases of Crimean-Congo hemorrhagic fever (CCHF) among humans in southern and western Mauritania, 2022. The departments (“moughataas” in the local Arabic dialect) and regions (“wilayas”) are shown on the map. The moughataas from which CCHF virus–positive patients originated are shown.

During the 2022 outbreak, 88 blood samples were collected from suspected individuals for laboratory confirmation of diagnosis. CCHF was confirmed by RT-PCR in 7 of 88 (8%) patients. Serum samples were not taken during the recovery period due to refusal of the patients. The mean age was 47 years (standard deviation, 19 years; range, 25–80 years), and the sex ratio (male/female) was 1:3. All confirmed cases had an agropastoral activity, and the majority (6/7) came from rural areas, with the exception of 1 butcher living and working in Nouakchott. The majority (6/7 [86%]) of the patients reported a history of tick bites few days preceding the onset of the initial symptoms. They reported repeated contact with ticks. Among the 7 CCHFV-positive patients, 4 (57%) were residents in the region (“wilaya”) of Trarza (Table 1, Figure 1).

**Table 1.** Characteristics of Crimean-Congo Hemorrhagic Fever Virus–Seropositive Individuals

No.	Date	Age	Sex	Wilaya^a^	Moughataa^b^	Profession	Agropastoral Occupation	Animal Contact	Tick Contact	Outcome
1	4 Feb 2022	52	M	Hodh El Gharbi	Kobeni	Breeder	Yes	Yes	Yes	Cured
2	11 Feb 2022	34	M	Hodh El Gharbi	Tintane	Breeder	Yes	Yes	Yes	Cured
3	14 Feb 2022	25	M	Trarza	Ouad Naga	Shepherd	Yes	Yes	Yes	Cured
4	14 Feb 2022	41	M	Nouakchott	Dar naim	Butcher	Yes	Yes	No	Died
5	21 Feb 2022	80	F	Trarza	Rkiz	None	Yes	No	Yes	Died
6	11 Mar 2022	35	F	Trarza	Boutilimit	None	Yes	Yes	Yes	Cured
7	24 Apr 2022	62	F	Trarza	Boutilimit	None	Yes	Yes	Yes	Cured

Abbreviations: F, female; M, male.

a “Wilaya” is a local term that refers to region.

b “Moughataa” is a local term that denotes province or department.

All patients who were positive for CCHFV had bleeding symptoms and had been taken to hospital rapidly. For all of these patients, their ELISA results were negative for all other tested viruses, namely Rift Valley fever, yellow fever, West Nile, dengue, and chikungunya. The affected patients were cattle breeders or shepherd (n = 3), butcher (n = 1), or housewives with agropastoral activities (n = 3). Among these 7 patients, there was no epidemiological or family link, and they lived in separate localities. No other members of the families of CCHFV-positive patients were affected, and they remained asymptomatic. The risk factor found in this study was contact with ticks (86%). Blood samples taken from patients after the onset of clinical signs were positive for CCHFV RNA. The interval between the onset of the initial symptoms and hospital admission was 2–3 days. Five of 7 patients were admitted to 1 of the collaborating hospitals in Mauritania (Centre Hospitalier de Kiffa, Hôpital Hamed, Centre Hospitalier Cheikh Zeid, Centre Hospitalier National de Nouakchott, and Hôpital de l’Amitié) and were treated promptly by blood transfusion as soon as they were admitted, and they were cured. Two other patients, who were referred late to the hospital, died.

## DISCUSSION

CCHF is a viral disease that is asymptomatic in infected animals but may cause serious health problems in humans [17]. The areas where many of our patients (6/7) were infected by CCHFV are characterized by vast plains irrigated by water from dams. The main activities of the population are agriculture and animal husbandry. These rural localities offer all the characteristics conducive to disease transmission. Moreover, climate changes in recent decades have led to a rise in the distribution of this virus [18]. This may be one of the reasons why no epidemics have been observed in urban areas. However, healthcare-related transmission of CCHF has been reported in hospitals and health centers and occurs in both high- and low-resource settings [19]. The failure to recognize CCHF and implement appropriate prevention and control procedures would result in a considerable nosocomial risk, especially in the context of critical care [20].

CCHFV is maintained in vertical and horizontal transmission cycles involving ixodid ticks and a variety of wild and domestic vertebrates, which do not show any sign of illness. The virus circulates in a number of tick genera, but *Hyalomma* ticks are the principal source of human infection, probably because both immature and adult forms actively seek hosts for blood meals required at each stage of maturation [21, 22]. CCHF occurs most frequently among agricultural workers following the bite of an infected tick, and to a lesser extent among slaughterhouse workers exposed to the blood and tissues of infected livestock and medical personnel through contact with the body fluids of infected patients [21]. Most livestock transhumance occurs during dry season and is one of the causes of the distribution of ticks in the endemic regions in Mauritania. In this investigation, the majority of the patients reported a history of tick bites in their place of residence, although all of them knew the tick and had repeated contact with the vector. Six patients had a history of tick bite <7 days before hospitalization. The high abundance of ticks and high infestation of livestock can be important causes of the CCHF epidemic. Most of the patients were male, suggesting a relationship between the disease and occupation in the rural areas. This corresponds to the presence of risk factors for CCHF [23].

Clinical diagnosis alone is difficult to establish due to the nonspecific nature of symptoms. Therefore, laboratory diagnosis is necessary for patients residing in or travel to CCHFV-endemic regions in whom the disease is suspected [24]. The incubation period is short with the onset of symptoms in generally less than a week. The initial symptoms are common to other infectious syndromes with fever, headache, myalgia, and gastrointestinal symptoms. The hemorrhagic syndrome occurs during a second phase with sometimes major bleeding in and from the mucous membranes and the skin [25]. In the outbreak reported in the present work, the interval between the onset of symptoms and hospital admission was only 2–3 days. For this reason, none of the cases received ribavirin therapy.

During the dry season (November to June), the movement of livestock and herds is intense between different regions, and the tick vectors move along with livestock. The virus can be transmitted between animals and humans. The movements of fauna and populations and nomadic pastoral practice could explain the progressive extension of the CCHFV in the southern regions of the country. This explains the notification of cases even in Nouakchott, the capital city of Mauritania. Two of the patients included in this study transported their cattle to Nouakchott to sell them at the city market. The virus could have circulated with viremic animals transported by truck between various grazing areas, including the region of Hodh El Gharbi toward Nouakchott and to other regions in southeastern Mauritania where CCHF is endemic. However, because of the small sample size of the present study, further epidemiological studies would be required in Mauritania, as well as in other countries, to assess the association between CCHF and various risk factors [26, 27].

## CONCLUSIONS

In the absence of medical prophylaxis and effective means of combating the natural reservoir, the prevention of CCHF requires measures of epidemiological surveillance and rapid response to outbreaks in animals or humans to avoid the spread of the disease. It is therefore necessary to implement the means to inform the population and caregivers (medical personnel and veterinary personnel) in endemic and enzootic areas about the disease and its prevention. Similarly, it would be necessary to have information tools that would quickly inform physicians and the population in endemic areas in the event of a resurgence of infections. It would be necessary to strengthen the capacities of the diagnostic laboratory for hemorrhagic fevers, to set up an active surveillance system, especially during the rainy seasons, and to carry out large-scale human and animal serological surveys to assess the prevalence of CCHF in the country.

## Notes

***Acknowledgments.*** We acknowledge the tireless effort of the physicians at the Centre Hospitalier de Kiffa, Hopital Hamed, Centre Hospitalier Cheikh Zeid, Centre Hopitalier National de Nouakchott, and Hopital de l’Amitié. We also thank the Regional Disease Surveillance Systems Enhancement, Institut National de Recherche en Santé Publique, the authorities of the Ministry of Health of Mauritania, in particular the Direction Générale de la Santé Publique, Direction de l’Information Stratégique et de la Surveillance Epidémiologique, and field agents, for facilitating the investigation of this outbreak.

***Financial support.*** None.

***Patient consent*.** It was not possible to obtain informed patient consent from 8 patients admitted to different hospitals in February to May 2022 in Mauritania with suspected Crimean-Congo hemorrhagic fever (CCHF). Their clinical course and outcomes were not described in this work. Written informed consent was obtained from individuals who were potentially in contact with those 8 patients with suspected CCHF. All information was anonymized. This work was reviewed and approved by the Institutional Review Board (Ethics and Scientific committee of the Medical Center of Kiffa) and the Mauritanian Ministry of Health.
